# Trends in Food Pathogens Risk Attenuation

**DOI:** 10.3390/microorganisms11082023

**Published:** 2023-08-06

**Authors:** Elisabeta Elena Popa, Elena Loredana Ungureanu, Mihaela Geicu-Cristea, Amalia Carmen Mitelut, Mihaela Cristina Draghici, Paul Alexandru Popescu, Mona Elena Popa

**Affiliations:** 1 Faculty of Biotechnology, University of Agronomic Sciences and Veterinary Medicine of Bucharest, 59 Marasti Blvd., 011464 Bucharest, Romania; mihaela_geicu@yahoo.com (M.G.-C.); amaliamitelut@yahoo.com (A.C.M.); mihaeladraghici38@gmail.com (M.C.D.); paul.alex.popescu@gmail.com (P.A.P.); pandry2002@yahoo.com (M.E.P.); 2 National Research and Development Institute for Food Bioresources, 6 Dinu Vintila Str., 021102 Bucharest, Romania

**Keywords:** food pathogens, foodborne illnesses, emerging technologies, antimicrobial packaging materials

## Abstract

Foodborne pathogens represent one of the most dangerous threats to public health along the food chain all over the world. Over time, many methods were studied for pathogen inhibition in food, such as the development of novel packaging materials with enhanced properties for microorganisms’ growth inhibition (coatings, films) and the use of emerging technologies, like ultrasound, radio frequency or microwave. The aim of this study was to evaluate the current trends in the food industry for pathogenic microorganisms’ inhibition and food preservation in two directions, namely technology used for food processing and novel packaging materials development. Five technologies were discussed in this study, namely high-voltage atmospheric cold plasma (HVACP), High-Pressure Processing (HPP), microwaves, radio frequency (RF) heating and ultrasound. These technologies proved to be efficient in the reduction of pathogenic microbial loads in different food products. Further, a series of studies were performed, related to novel packaging material development, by using a series of antimicrobial agents such as natural extracts, bacteriocins or antimicrobial nanoparticles. These materials proved to be efficient in the inhibition of a wide range of microorganisms, including Gram-negative and Gram-positive bacteria, fungi and yeasts.

## 1. Introduction

Many foodborne disease outbreaks were reported over the years in different food products which were contaminated with microorganisms, especially pathogenic ones [1]. For example, in the United States (U.S.), approximately 9.4 million people a year develop foodborne illnesses which are caused by known pathogens [2]. In Europe, in 2021, a total of 4005 foodborne outbreaks were reported by EFSA (European Food Safety Authority) [3]. The main pathogens that cause foodborne illnesses are *Salmonella* spp., *E. coli* O157:H7 and O121, *Listeria monocytogenes*, *Cyclospora* and *Vibrio parahaemolyticus* [4].

Consumers are increasingly asking for pre-cooked foods, a fact that led to great research in the area of food safety, especially from a microbiological point of view [5]. Much research has been conducted in order to overcome the pathogenic microorganisms’ threats to human health, especially due to the fact that they could become resistant to treatments [6], raising the rates of morbidity and mortality [7].

Foodborne illnesses are generally caused by the consumption of food products contaminated with microorganisms or their toxins, contamination that can occur during any step along the food supply chain [8].

Therefore, there is an increasing interest in obtaining safe food products, from all points of view. In this respect, many studies have been performed in order to develop methods for pathogenic microorganisms’ reduction or inactivation in food. The aim of this study was to evaluate the current trends in pathogen risk attenuation regarding the emerging technologies used for food preservation as well as novel food packaging materials with enhanced antimicrobial properties.

## 2. Emerging Technologies for Microbial Inactivation

### 2.1. High-Voltage Atmospheric Cold Plasma (HVACP)

High-voltage atmospheric cold plasma (HVACP) is a novel technology that gained more attention in recent years, showing promising results in respect to microbial inactivation under low temperatures [9]. The inactivation mechanism of HVACP reported in the literature is related to reactive gas species, such as reactive oxygen species and reactive nitrogen species, and some contribution from ultraviolet light [10], which are known to be agents that causes stress and oxidation in microbial cells [11]. It consists of electrons, ions and elements in their fundamental and excited states, which are generated when a high-voltage electric field is applied into any gas at atmospheric pressure and room temperature [12,13]. These agents lead to the damaging of the cell membrane, surface etching and electroporation, which finally leads to microbial cell damage and/or death [14]. This technology can be used in the treatment of packed food products and achieves a great reduction in food spoilage microorganisms, including foodborne pathogens, maintaining the quality of food products at the same time. Han et al., (2016) [15] demonstrated that the effectiveness of HVACP depends on process and system parameters, such as treatment time or post-treatment storage time. These parameters influenced the quantity and duration of reactive species, which played a crucial role in the antimicrobial reactions. Wan et al., (2019) [9] studied the inactivation of *Listeria innocua* inoculated on tryptic soy agar, Queso Fresco cheese and a cheese model by treatment with HVACP for 5 min. It was shown that direct treatment led to a reduction of 5.0 (tryptic soy agar), 3.5 (Queso Fresco) and 1.6 (cheese model) log CFU/g, being more effective compared to indirect treatment. Further, Wan et al., (2021) [16] inoculated Queso Fresco with *L. innocua* and *E. coli* K-12 and then treated the samples with HVACP for up to 5 min at 60, 80 and 100 kV. The sample treated for 5 min at 100 kV in dry air showed a reduction of 1.4 log CFU/g of *L. innocua* and 3.5 log CFU/g for *E. coli* K-12 after storage for 24 h at refrigeration temperatures. The results showed that by increasing the voltage from 60 to 100 kV, the reduction in microbial load increased as well. Furthermore, the HVACP treatment resulted in minimal alterations in the pH, color, and lipid oxidation of Queso Fresco and no significant (*p* ≥ 0.05) changes in texture, with the crumbliness of the cheese remaining unaffected after plasma treatment. On the contrary, Cai et al., (2022) [17] highlight that the hardness value of the treated samples consistently exceeded that of the control group, because HVACP treatment can accelerate protein oxidation, which can help inhibit enzymatic degradation and prevent a decrease in hardness. Also, Ott et al., (2022) [18] investigated the inactivation of *L. monocytogenes* in Queso Fresco cheese by using HVACP treatment for 0, 1, 2 and 3 min. The results showed that after 1 min of treatment, a significant reduction in *L. monocytogenes* was observed, and the reduction increased as the exposure time increased. Furthermore, microbial enumeration was performed on in 1 g, 10 g and 100 g of sample, showing a mass dependency of microorganism reduction, concluding that a higher mass of product should be treated with a prolonged exposure.

Another study conducted by Wang et al., (2022) [19] investigated the effect of HVACP on various microorganisms from fresh tilapia fillets. Further, they treated the samples for 1, 3 and 5 min at 70 kV, and then the samples were stored at refrigeration temperatures and analyzed during storage. The 5 min treatment showed a good reduction in all determined bacteria. After 12 days of storage, significantly lower loads were registered for of total viable bacteria (7.15 log CFU/g), *Pseudomonas* spp. (6.99 log CFU/g) and *Enterobacteriaceae* (4.23 log CFU/g), compared to control samples.

Illera et al., (2022) [13] aimed to use HVACP technology to inactivate previously inoculated *Salmonella enteritidis* on the surface of disinfected egg shells. The treatment conditions were: 100 kV, 60% air humidity and 1 min treatment and six hours post-treatment or, alternatively, five minutes of treatment and four hours post-treatment. The results showed variable values in *Salmonella* reduction when combining the studied conditions for treatment and posttreatment; however, although a certain number of colonies that were injured but still viable still remained, the applied treatments presented a lethal effect on *S. enteritidis* cells. The microbial reduction was influenced also by post-treatment time and applied voltage. Therefore, an increasing post-treatment time from 0 to 24 h led to an increasing reduction in *S. enteritidis* with 2.45 log, regardless of the other parameters. A similar effect was observed when increasing the voltage from 80 to 100 kW, with a further reduction in *S. enteritidis* of 2.27 log. Mahnot et al., (2019) [10] investigated the efficiency of HVACP technology on *Salmonella enterica* serovar Typhimurium LT2 inactivation in tender coconut water. The results showed that a treatment of 120 s at 90 kV led to a reduction of 1.30 log of *Salmonella* and a reduction of 5 log was achieved when 400 ppm citric acid was added, demonstrating that this technology could be further used in fruit and vegetable juice preservation.

Sudarsan and Keener (2022a) [14] investigated HVACP technology as a potential treatment for microorganisms’ inactivation in food. They treated baby spinach leaves using high purity nitrogen gas and investigated the effects of the treatment on the native microbiota of the samples after 7 days of storage at 4 °C. A reduction up to 2.6 log CFU/sample was registered for 2 min treatment and 3.5 log CFU/sample for 5 min treatment at the end of the storage period. Furthermore, baby spinach leaves were inoculated with *S. enterica* serovars and *E. coli* 25,922 by Sudarsan and Keener (2022b) [11], and then treated with HVACP for 5 min at high humidity and 80 kV (indirect treatment). After 14 days of refrigerated storage a reduction of 3.18 log CFU/sample was observed in *S. enterica*, and for *E. coli*, it was determined a reduction of 3.77 log CFU/sample.

Liu et al., (2022) [20] also used HVACP to investigate its effects on *Pseudomonas aeruginosa* inactivation. A treatment at 75 kV for 90 s proved to be efficient, increasing the mortality rate from 48.16% to 94.45%, preventing biofilm formation this way.

HVACP technology has been used for product surface decontamination of various microorganisms in packed products [21], presenting great results with short exposure times, minimal inputs of air and electricity, no heating effects and with a minimal water consumption [12]. The advantages presented by this technology for the food industry are the short processing time (from few seconds to few minutes), operational simplicity [19], a high efficiency at room temperature, being suitable for heat-sensitive products, its operation at atmospheric pressure and, in terms of sustainability, it having low energy requirements [12,22,23,24]. However, this technique has some drawbacks, including protein oxidation, lipid oxidation, alterations in organoleptic properties, discoloration of food products [25] and the non-uniformity of the treatment [26].

### 2.2. High-Pressure Processing (HPP)

High-Pressure Processing (HPP) represents a method for food processing in which food products are subjected to high pressure (100–1000 MPa) [27]. It is a non-thermal pasteurization technology that has fewer negative effects on food quality compared to conventional thermal processes [28,29,30]. In industry, HPP is generally used at 400–600 MPa for liquid and solid food products and the treatment time is from 3 to 10 min [29,31]. The mechanism of action of HPP is related to the many changes that this treatment induces in the microbial cell, like inhibition of protein synthesis or of key enzymes, as well as changes in the cell morphology and disruption of genetic mechanisms [27,32]. However, the inactivation of microorganisms through HPP is influenced by various factors, such as the pressure magnitude, holding time, process temperature, compression and de-compression rates, the microbiota, and the intrinsic properties of food products [27].

Ferreira et al., (2023) [32] investigated the effect of HPP technology on *Latilactobacillus sakei* inactivation in a meat emulsion model, by varying pressure (400–600 MPa) and time of exposure (180–480 s). The results of the study showed a reduction in *L. sakei* from 0.99 to 4.12 UFC/g function in the applied conditions. When higher pressure treatments were applied, the treatment was more effective. Similarly, tilapia fillets were treated at 100, 200, 300 or 400 MPa by Seumitsu and Cristianini (2019) [33] in order to evaluate the effect of HPP on their quality. It resulted in a significant reduction in psychrotrophic microorganisms in samples treated at 300 and 400 MPa, of 1.11 CFU/g and 1.15 CFU/g, respectively. Beef steaks inoculated with *E. coli* 0157:H7, vacuum packed and treated by HPP, were investigated by Sun et al., (2017) [34]. It resulted that a treatment of 15 min at 450 MPa effectively inactivated 4.74 log CFU/g of *E. coli*. Furthermore, a treatment of 600 MPa for 10 min resulted in 6.13 log CFU/g microbial reduction.

Pokhrel et al., (2022) [35] investigated the effect of HPP treatment (200, 300, 400 MPa for 1–5 min) on microbial inactivation of carrot–orange juice mixtures. A reduction in *L. innocua* higher than 6 log was determined when using HPP treatments at 300 MPa for 2 min and 400 MPa for 1 and 3 min. Furthermore, the natural microbiota of the tested juices remained under 2 log CFU/mL for a period of 28 days of storage.

Coconut water was subjected to HPP treatment by Raghubeer et al., (2020) [36] in order to inhibit pathogens and spoilage microbiota. Therefore, samples were inoculated with *E. coli* O157:H7, *Salmonella* spp. and *L. monocytogenes* in form of multiple strain mixtures, and a 593 MPa HPP treatment was applied for 3 min at 4 °C. Further, the obtained samples were stored at refrigeration temperatures for 54 and 75 days. The results showed that at the end of storage period, samples treated with HPP presented less than 1 or no CFU/mL compared to control samples for which 3 log/mL were determined for the pathogenic mixture used. During the storage period of 120 days, microbial counts were of about 2 log CFU/mL for uninoculated samples with no detection of any microorganisms for HPP-treated samples.

In their study, Usaga et al., (2021) [37] investigated HPP treatment as a method to inactivate *E. coli* O157:H7, *S. enterica* and *L. monocytogenes* in acid and acidified juices and beverages. A 550 MPa treatment for 1 min resulted in more than 5 log reduction for all tested microorganisms.

Woldemariam et al., (2022) [38] performed a study with the aim of determining the inactivation of naturally occurring microorganisms by HPP (100–600 MPa for 30–600 s) in red pepper paste. The aerobic mesophilic bacteria count was reduced by 4.5 log CFU/g at 527 MPa for 517 s and yeast count was reduced to 1 log CFU/g at 600 MPa for 315 s.

Because HPP affects various factors, including cell membrane permeability, alterations in cell morphology, changes in biochemical reactions, and interference with genetic mechanisms within microorganisms, microbial cell death occurs due to the simultaneous effects that impact either less critical components or critical parameters [39].

This method significantly affects the texture of food by modifying structural components like proteins and influencing enzyme activity [40]. The majority of studies have indicated that high pressure (HP)-treated juices and smoothies exhibit sensory profiles closer to fresh samples rather than samples treated by traditional processing, most probably due to the inactivation of several oxidative and pectic enzymes induced by HP, along with the limited impact on the structure of small molecular flavor compounds. However, these findings are influenced by the type of food products treated, sensory evaluation methods, and the assessors involved [41]. High hydrostatic pressure processing is recognized for its limited influence on the nutritional and chemical composition of the foods [40], but also for its minimal impact on smaller molecules such as volatile compounds, pigments, vitamins and other compounds associated with sensory, nutritional and health-promoting effects [42]. HPP offers several advantages, including reduced process times, minimal heat penetration or heat damage issues, and preserved freshness, flavor and color of the products. It also prevents vitamin C loss and minimizes functionality alterations compared to traditional thermal processing methods. Furthermore, HPP results in an extended shelf life, enabling wider product distribution and reducing product returns. Additionally, HPP uses less energy, leading to lower greenhouse gas emissions, and it boasts the highest processing efficiency for pumpable foods. The process can be conducted in the final packaging, avoiding post-processing contamination and tempering for retail processors. Moreover, HPP reduces required processing times and eliminates the generation of by-products. It allows for the inactivation of microorganisms and enzymes at low temperatures, while preserving valuable low molecular constituents, such as bioactive substances, vitamins, colors and flavorings. In addition, there are certain challenges of HPP, such as heat transfer problems leading to non-uniform processing, the need for reliable and reproducible data for process validation, limited understanding of the interaction between high pressure and various food constituents, and packaging concerns [43].

### 2.3. Microwave (MW)

Microwave processing is a novel technology that combines thermal and non-thermal effects [44] and has been used as a method of microorganism’s inactivation in food products (solid and semi-solid), liquids and surfaces [45,46]. During this process, the product is exposed to heat for a shorter time compared to conventional treatment, and the target microorganisms are inactivated faster, keeping a low quality loss [32,45] and having a higher energy efficiency compared to conventional heating [47]. The dielectric properties, size, shape, packaging materials, orientation of food in relation to the oven, physical state of water in the product, the presence of bone in a meat product, and moisture content are factors that influence microbial inactivation by MW [48]. The mechanism of action of microwave treatment is generally related to the thermal effect that leads to the inactivation of microorganisms [49]. However, non-thermal effects of MW (below 40 °C) have been shown to be more effective in destroying microorganisms. Low-energy and short-term MW irradiation at sublethal temperatures (40 °C) can impact the permeability of bacterial cell membranes. On the other hand, high-energy and long-term MW irradiation can cause irreversible damage to the bacterial membrane and cytoplasmic membrane, directly leading to bacterial death [50].

Salmon fillets inoculated with *Clostridium sporogenes* were treated using a microwave processing system at different radiation times (2, 3, 4 min) and different holding times (0, 2, 4 min) by Guo et al., (2020) [44]. The results showed that presenting same or lower thermal lethality values, all the experimented microwave treatments led to a higher reduction in *C. sporogenes* compared to their corresponding water bath processes.

Siguemoto et al., (2018) [51] researched the inactivation kinetics of two pathogenic bacteria, namely *E. coli* O157:H7 (CECT 4972) and *L. monocytogenes* (CECT 4032), previously inoculated in apple juice using microwave processing (400, 600, 800 and 1000 W) in comparison with conventional isothermal treatment (55, 60, 65 and 70 °C). The results showed the possibility of reaching 5 log reduction for both treatments, with the microwave treatment being more efficient than predicted, suggesting an enhanced inactivation of this process. Also in juices, Mendes-Oliveira et al., (2020) [52] aimed to determine the inactivation kinetics of *E. coli* O157:H7 and *S. typhimurium* by microwave processing at 80–90 °C (conditions generally met in conventional pasteurization). Therefore, different power levels (600 W, 720 W) were used to treat inoculated juices for 5 s, 10 s, 15 s, 20 s and 25 s. The results of the study showed that inactivation levels increased with an increasing treatment power, temperature and time, achieving a reduction of up to 7 log. Furthermore, Ergün et al., (2021) [53] aimed to determine the thermal resistance of *Zygosaccharomyces rouxii* in apricots and figs using pasteurization by microwave processing. The results showed a 5 log reduction of *Z. rouxii* in both tested fruits.

The advantages of MWs include simple operation, reduced invasiveness, deep penetration depth, local controllability, and a wide heating area [50], shortening the heating time and energy efficiency due to the shorter processing time [54]. The main drawback of microwave heating is the uneven distribution of temperature, resulting in cold and warm zones, which can lead to incomplete deactivation of microorganisms [55]. Also, the varying of food composition can influence the mode of microwave heating and its effects on the inactivation of foodborne pathogens [56]. Cho and Chung (2020) [54] demonstrated that the sensory characteristics of the tested food, including taste, flavor, color and texture, showed a significant improvement (*p* < 0.05) after microwave treatment compared to conventional conduction heating. Microwave heating can impact the nutritional quality of food by influencing the interactions of starch, fat and protein under these conditions. Specifically, microwave treatment of free fatty acids in food can lead to an increase in their content and alter their composition. Monounsaturated fatty acid (MUFA) and polyunsaturated fatty acid (PUFA) proportions decrease, while saturated fatty acid (SFA) and trans fatty acid (TFA) proportions increase. Furthermore, microwave treatment can bring about changes in the structure of proteins, affecting various properties such as hydrophobicity, digestibility, emulsification, foaming, gel resistance, oxidation and allergenicity. It can also impact the Maillard process between proteins and decreasing sugars. Additionally, microwave treatment can enhance the antioxidant ability of proteins by promoting protein hydrolysis, resulting in the production of more active peptides and an improvement in the metal chelating ability of proteins [57].

### 2.4. Radio Frequency (RF) Heating

Radio frequency (RF) heating uses electromagnetic energy [58,59] at frequencies between 1 and 300 MHz, but in industrial, scientific and medical applications, only values of 13.56, 27.12 and 40.68 MHz are used [59]. It generates heat inside food products through ionic conduction and dipole rotation, giving fast heating at a volumetric level all over the food product [60,61]. It is one of the most efficient technologies for pasteurization of food products, and is also environmentally friendly [62].

The mechanism of microbial inactivation using RF has been primarily attributed to heat. However, a nonthermal effect was introduced in 2008, suggesting that exposure to RF radiation can lead to changes in the structure of the bacterial membrane. This can result in injury and the subsequent leakage of intracellular components, including ATP, nucleic acids, and proteins. Such leakage disrupts the energy system and enzymatic activity, ultimately leading to cell death [63].

Rincon and Singh (2016) [64] aimed to evaluate the thermal inactivation of *E. coli* (O157:H7, O26:H11 and O111) producing Shiga toxin and non-pathogenic *E. coli* by RF heating in non-intact beef steaks. The steaks were inoculated, individually vacuum packed and treated with RF (27.12 MHz) in order to obtain rare (60 °C) and medium-rare (65 °C) steaks. Following the treatment, a reduction in microbial load was observed, namely 0.99 log CFU/g for *E. coli* O157:H7, 3.08 log CFU/g for *E. coli* O26:H11, 2.85 log CFU/g for *E. coli* O111 and 5.0 log CFU/g for the non-pathogenic strain in the case of a treatment temperature of 60 °C, while for treatment at 65 °C, a 5 log CFU/g reduction was achieved for all strains. Further, the authors took into consideration a middle temperature of 63 °C, excluding the non-pathogenic strain based on its demonstrated sensibility. The applied treatment resulted in a 5 log CFU/g reduction for *E. coli* O111 and *E. coli* O157:H7, but not for *E. coli* O26:H11. The authors concluded that RF treatment at 65 °C represents an efficient way to reduce the microbial load of the tested *E. coli* strains.

Zhang et al., (2020) [65] investigated the use of RF heating for inactivation of *Cronobacter sakazakii* in powdered formula milk for infants. The study showed that the microbial load was reduced when the temperature was increased from 55 to 70 °C with a value of a_w_ of the sample of 0.2–0.4. However, a combination of RF and hot air pasteurization was more efficient in *C. sakazakii* inactivation.

Rice samples with different milling degrees inoculated with *S. typhimurium* and *Staphylococcus aureus* were subjected to RF heating for 0 to 75 s. Pathogen reduction was observed to be higher in rice with a milling degree of 0 and 2%, compared to a milling degree of 8 and 10% using the same treatment condition. Also, differences in heating rates showed significant differences in microorganism inactivation [66]. RF treatment was also proven to be efficient in the inactivation of *Bacillus cereus* in buckwheat kernels at 105 °C for 30 min, as demonstrated by Xu et al., (2023) [67].

Xu et al., (2022) [68] investigated RF heating of plant essential oils, in combination or alone for the inactivation of inoculated *Salmonella* and natural microflora in sesame and flax seed. Oregano oil and cinnamon oil were both applied as treatment for 7 days on the studied oils and proved to be highly efficient in the inactivation of *Salmonella*, with better results obtained for cinnamon oil. A combination of RF heating (at 80 °C and 85 °C) and cinnamon oil at 0.83 µL/mL for 3 days let to a reduction of more than 5 log CFU/g on both inoculated seeds. However, the reduction in total bacterial counts was only of 0.96 log CFU (sesame seeds) and 1.42 (flax seeds), while for yeast and mold, a reduction of 1.05 and 1.56 log CFU/g was registered.

Wei et al., (2018) [69] aimed to investigate RF treatment effects on the inactivation of *S. enterica* and *Enterococcus faecium* in black peppercorn. An efficient RF treatment was proved by a heating time of 2.5 min with a 5.31 log CFU for *Salmonella* and 5.26 log CFU/g reduction for *E. faecium* in the entire sample contained by the tray. RF treatment was similarly applied for inactivation of *S. typhimurium* in red pepper powders by Hu et al., (2018) [70]. The authors observed that at lower values of a_w_ (0.57–0.71), the heating rate increased, but decreased when a_w_ value reached 0.71, achieving a 2–3 log reduction depending on the values of a_w_ (the best reduction results being registered for the samples with 0.71 a_w_). Therefore, an RF treatment at 70 °C (with 110 s until reaching the value and a holding time over 60 s) could reach a more than 5 log reduction of *S. typhimurium* in red pepper powders with an a_w_ value of 0.71. By taking into account the a_w_ values, Jiao et al., (2019) [71] also studied red pepper powder treated with RF for the inactivation of *B. cereus* spores. As obtained by Hu et al., (2018) [70], the heating rate increased with the increasing sample’s a_w_ (from 0.56 to 0.74), but decreased when the sample reached a value of 0.70 for a_w_. The results of the study showed an optimum inactivation effect of RF of approximate 4 log reduction when the sample having a_w_ of 0.70 was heated at 90 °C (in 110 s) with a holding time of 12 min. In another study, black pepper kernels were inoculated with *E. coli* O157: H7 and *S. typhimurium* and treated by RF by Tong et al., (2022) [72] in order to determine the inactivation kinetics of the tested microbial strains. The results showed a reduction of more than 6 log for *E. coli* O157: H7 (at 90 °C heating temperature for 7 min) and *S. typhimurium* (at 100 °C heating temperature for 8 min), proving the efficacy of studied strains’ reduction of RF treatment.

The successful application of RF heating for food products relies on several factors, including dielectric properties, frequency, food properties and temperature, food composition, and density. These factors play a crucial role in the effectiveness and efficiency of RF heating in the food industry [24]. RF technology offers several advantages, including a longer wavelength, no direct contact between the electrode and food, a straightforward construction design, and higher energy efficiency, resulting in improved final product quality. It is well-suited for industrial applications, especially for processing liquid and solid foods. However, this technology also comes with some drawbacks, such as higher equipment and operational costs, lower power density, slower heating rates, and a potential impact on output quality [24].

RF technology impacts the texture of food by modifying the network structure of proteins. Moreover, changes in texture can be attributed to alterations in the microstructure of tissue cells and changes in the chemical composition [73]. Also, this method provides a higher preservation of the sensory and nutritional qualities of food due to its relatively better heating uniformity and deeper penetration [74].

### 2.5. Ultrasound (US)

Ultrasound (US) technology is used either at 20–100 MHz (low frequency ultrasound) or above 100 MHz (high frequency ultrasound), and has been reported to be efficient in microbial inactivation [75,76], and is also considered a green technology [77,78]. The microbial inactivation by using US technology is mainly influenced by cavitation thresholds (external pressure, temperature, frequency, amplitude and intensity), media (initial microbial number, pH, volume and viscosity), and type of microorganism (growth phases, spores or vegetative cells, size and shape and cell wall) [79]. The mechanism of microbial inactivation by ultrasound is believed to involve several factors, including cell membrane thinning, high localized temperature, and the generation of free radicals. The main bactericidal effect of ultrasound is associated with changes in pressure inside the medium [63]. During high frequency ultrasound treatment, cavitation bubbles are generated and they are known to inactivate enzymes and produce damage to membranes, proteins, DNA (deoxyribonucleic acid) and RNA (ribonucleic acid) by the generation of physical and chemical effects [75]. However, the effects of US treatment depend on intensity (applied power), media, processing time and the temperature of food products when a constant wave frequency is maintained [80], as well as the type of targeted microorganism [81].

Esua et al., (2022) [82] evaluated the linear and non-linear models for inactivation kinetics prediction in respect to *E. coli* and *L. monocytogenes* in grass carp by applying a treatment of combined ultrasound and plasma functionalized buffer. The results of the study showed that the combined treatment presented reductions of 3.92 log CFU/g for *E. coli* and 3.70 log CFU/g for *L. monocytogenes*, which was higher than the treatments alone.

Ma et al., (2023) [83] studied the inactivation of *Vibrio parahaemolyticus* and microbial growth on raw oysters by using US treatment. A reduction of 3.13 log CFU/g was registered for *V. parahaemolyticus* after being treated for 12.5 min by 7.5 W/mL. Furthermore, total microbial growth was retarded following the applied treatment, prolonging the shelf life of the studies oysters.

A combination between bacteriocin thurincin H and power ultrasound was investigated by Anda et al., (2022) [84] for the inactivation of *E. coli* K-12 and *L. innocua* ATCC 33090 in milk and orange juice. Therefore, 40 µg/g of thurincin H and a treatment of power ultrasound at a frequency of 20–25 MHz and a temperature of 30 ± 5 °C were applied on the studied products, and the results showed a higher efficiency against *E. coli* compared to *L. innocua* in milk, with more than 2.8 Log CFU/mL, while with regards to the orange juice, a reduction of 5.5 log CFU/mL was determined for *L. innocua* and 3.4 log CFU/mL for *E. coli*.

High intensity US was applied in the presence of salt (NaCl) and salt replacers (KCl) for inactivation of *E. coli* K12 and *L. innocua* suspensions at 20 or 33 kHz, alone or in combination, by Inguglia et al., (2018) [81]. A reduction of up to 6 log was registered for *E. coli* K12 and 4 log for *L. innocua*, regardless of the presence of NaCl or KCl.

The inactivation kinetics of *Aspergillus niger* and *Clostridium butyricum* spores by using a treatment of supercritical carbon dioxide intensified with high-power ultrasound was investigated by Gomez-Gomez et al., (2021) [85] at different temperatures (50 °C, 60 °C, 70 °C, 80 °C, 85 °C) and pressures (100, 350 and 550 bar). Supercritical carbon dioxide treatment was proved to be more efficient in the inactivation of *A. niger* than of *C. butyricum*. However, the application of high-power ultrasound combined with supercritical carbon dioxide intensified the inactivation of *C. butyricum*, reducing the total inactivation time to 3 min from 10 min.

Costello et al., (2021) [86] combined nisin and ultrasound in the investigation of *L. innocua* and *E. coli* inactivation at 44, 500 and 1000 kHz. The results showed that when using US, *L. Innocua* resisted all applied treatments, while *E. coli* was inactivated when using 500 kHz. When nisin and US was applied, no effect on *L. innocua* was observed, while an enhancement of *E. coli* inactivation was observed, but only at 500 kHz as well.

The emerging technologies possess the capability to create food products with im-proved nutritional, sensory, and safety attributes compared to conventional thermal processes. Nevertheless, the implementation of these nonthermal technologies in the food industry is constrained by the substantial initial setup cost and the need to modernize existing processing lines [87].

Novel and innovative food processing techniques aim to achieve key sustainability objectives by minimizing the environmental impact of food processing through waste reduction and the efficient use of natural resources such as energy and water. These techniques also strive to provide safe, nutritious, high-quality products for consumers. Achieving sustainability in food production systems demands a comprehensive approach that considers the entire supply chain, from production to the end product.

Consumers may not readily recognize the technological, economic, social and environmental benefits, as well as perceived risks, associated with a new technology. While some consumers prioritize environmental sustainability when assessing new technologies and prefer processes with minimal or no technological interventions, others embrace innovation and believe that new technologies offer advantages and reduce risks [88].

In terms of sustainability, cold plasma has the ability to operate at low temperatures, resulting in minimal energy consumption, reduced processing time and effective microbial reduction. This technique is also compatible with most food products and does not negatively impact the food matrix. Additionally, its eco-friendliness is evident, as it does not require the use of water or other solvents in the treatment process [87]. While HPP may initially seem more expensive than traditional thermal pasteurization processes, it actually demonstrates a lower environmental impact across almost all impact categories. In comparison to Modified Atmosphere Packaging (MAP), HPP is not only less costly but also has a reduced impact in most of the impact categories. MAP often necessitates a substantial amount of packaging materials and food gases. Despite being a well-known non-thermal technology, HPP has had limited use in the industry, primarily due to the high electricity costs associated with the process [89]. Also, microwave heating technologies, particularly in food processing, have demonstrated that this low-energy consumption approach qualifies as a sustainable manufacturing technology. It aligns with sustainability-oriented and eco-friendly principles by reducing power consumption and minimizing environmental impact. The compact design of microwave equipment and lower overall operating costs make it an appealing alternative to traditional heat treatment methods and systems [90]. RF processing is a sustainability-oriented technology that finds wide application in various fields of human activity. The selectivity of RF enables the saving of input power. By utilizing only electrical energy, it reduces the reliance on fossil fuels in industry and the use of chemicals in agriculture [90]. The ultrasound technique aligns with green chemistry and eco-friendly characteristics. It serves as a sustainable alternative to the industry by avoiding the use of chemical solvents. Additionally, it has minimal impact on the sensorial and health-promoting attributes of food products. Compared to conventional processes, ultrasound offers several benefits in achieving sustainability goals. It reduces processing time and costs, simplifies manipulation, provides a higher purity of the final product, eliminates the need for post-treatment water waste, and consumes only a fraction of the energy and time required by conventional methods. This technology contributes to achieving the sustainable goals of food safety and security by reducing microorganisms and contaminants or causing changes in enzyme activities [91].

## 3. Novel Packaging Materials Used for Food Pathogens Inhibition

An antimicrobial packaging system can be achieved through the direct integration of antimicrobial agents into packaging films, applying coatings of these antimicrobial substances onto packaging films, or developing packaging materials using polymers. Typically, antimicrobial packaging systems are classified as either migrating or non-migrating, depending on the specific antimicrobial agent utilized and its interactions with both the packaging and food matrix. Antimicrobial films provide certain advantages over directly adding preservatives to food products. By applying preservative agents to the packaging material, only minimal amounts of preservatives come into contact with the food. This delivery mechanism ensures that the necessary amount of antimicrobials is used without directly incorporating it into the food product [92].

Antimicrobial packaging offers an additional benefit by mitigating the potential loss of antimicrobial activity that may occur when antimicrobials are directly added to food products. This loss can be attributed to leaching into the food matrix and interactions with other components like lipids and proteins. In contrast, antimicrobial packaging allows for controlled migration of the antimicrobial compound into the food, ensuring not only initial inhibition of undesirable microorganisms, but also sustained activity during the transportation and storage of food throughout the distribution process [92].

In order for a membrane to be classified as an edible film, it needs to meet the following criteria: (1) Sensory properties: the films should be transparent, not impart any distinct flavor or aroma to the food, and should go unnoticed during consumption; (2) barrier properties: they should possess suitable permeability to water vapor and solutes, while also exhibiting selective permeability to gases and volatiles; (3) mechanical properties: The films should be resistant to breakage and abrasion, enhancing the handling of food products. Additionally, they should be flexible enough to withstand deformations without fracturing; and (4) safety: edible films must be free from toxins and deemed safe for consumption. By reducing moisture and solute migration, respiration, gas exchange, and oxidative reactions, these films contribute to prolonging the shelf life of food products. Furthermore, they can potentially decrease or even prevent the occurrence of physiological disorders [93].

### 3.1. Biopolymers and Bio-Nanocomposites

Bio-based materials are eco-friendly and sustainable packaging materials that effectively prevent spoilage and disease by creating barriers against microorganisms and insects. They primarily rely on bio-polymers, which are highly valued in industries for their attractive properties like biocompatibility, chemical stability, and biodegradability. Common types of bio-based antimicrobial materials used in food packaging include carbohydrate (polysaccharide)-based materials, protein-based materials and lipid-based materials [94].

Polysaccharides like chitosan, starch, and cellulose are biodegradable and non-toxic materials. Some of them possess a semi-crystalline state and acid hydrolysis properties, making them promising sources for nanosized reinforcements due to their ability to release crystalline sections. Chitosan (CS), in particular, is extensively researched and utilized in food coating and packaging. It exhibits exceptional film-forming, antimicrobial and biodegradable properties. The antibacterial effectiveness of chitosan is influenced by factors such as pH value, molecular weight, and degree of deacetylation. Casted films made from chitosan demonstrate potent antibacterial activity, especially at lower pH levels, attributed to the protonated form of the amino group present in chitosan [94]. The antimicrobial properties of chitosan stem from its ability to bind to the cell surface through free amino groups. This interaction disrupts the integrity of the cell membrane, leading to cell death as a result of leakage of intracellular components [95].

Protein-based packaging materials, such as whey protein, soy protein, wheat gluten, zein, casein, collagen and gelatin, have undergone extensive research to explore their thermal, mechanical and barrier properties. These materials have been extensively studied due to their abundant resources, biodegradability, wide availability and their ability to control the release of additives and bioactive compounds, including antimicrobial agents, within the packaging system [94].

Bio-nanocomposites represent a novel generation of eco-friendly and high-performance materials for nano food packaging, which offer excellent antimicrobial properties, thereby extending the shelf life of food products and preserving their quality. Bio-nanocomposites consist of bio-based polymeric matrices reinforced with nanofillers or nanoparticles, resulting in superior characteristics compared to traditional bio-based polymers. Various types of antimicrobial bio-nanocomposites used in food packaging can be categorized based on the filler material with antimicrobial properties, including metallic-based bio-nanocomposites, clay and silicate-based bio-nanocomposites, nano cellulose-based bio-nanocomposites, and layered double hydroxide-based bio-nanocomposites. These innovative materials exhibit antimicrobial activity and demonstrate stability under high temperatures and pressures [94].

For this purpose, Musso et al., (2017) [96] found that the gelatin/curcumin films did not exhibit any antimicrobial activity against *S. enteritidis*, *E. coli*, *B. cereus*, and *S. aureus*. They attributed this lack of antimicrobial activity to two main factors. Firstly, the relatively low concentration of curcumin used (0.4 wt% based on gelatin) might have limited its effectiveness as an antimicrobial agent. Secondly, interactions between curcumin and gelatin could have played a role in reducing its antimicrobial properties in the film.

Mauro et al., (2022) [97] obtained a chitosan film that exhibited robust inhibition against *E. coli* (PSL52), *Hafnia paralvei* and *S. typhimurium*. Additionally, the film displayed noticeable inhibition against *E. coli* (ATCC 25922), *Stenotrophomonas maltophilia*, *Acinetobacter guillouiae*, *Hafnia alvei*, *S. enteritidis*, *Enterobacter amnigenus* and *P. aeruginosa*.

The bioactive films obtained by Trejo-Gonzalez et al., (2018) [98], composed of 1% (*w*/*v*) citrus pectin, 0.2% (*w*/*v*) gellan gum, 0.5% (*w*/*v*) glycerol and 5 mM CaCl_2_, revealed inhibitory effects against *L. monocytogenes*, *E. coli* and *S. aureus*. The authors consider that the antimicrobial activity observed in the FC films can be attributed to the presence of gellan gum and pectin.

Chitosan/gelatine emulsions and dispersions demonstrated superior antimicrobial efficacy compared to pectin/gelatine emulsions against Gram-positive bacteria (*S. aureus*) and Gram-positive spore-forming bacteria (*B. subtilis*), as well as exhibiting effectiveness against more resilient Gram-negative bacteria (*E. coli*) [99].

Starch-based nanocomposite films prepared by synthesizing chitosan nanoparticles (NPs) through ionic gelation, demonstrated superior antimicrobial efficacy against *B. cereus*, *S. aureus*, *E. coli* and *S. typhimurium*, compared to starch-based films [100].

#### 3.1.1. Biopolymers and Bio-Nanocomposites Enriched with Natural Antimicrobial Agents

In recent times, there has been a growing consumer preference for natural products as opposed to synthetic ones. As a result, naturally derived antimicrobial agents have gained significant importance in antimicrobial packaging. This is primarily due to the perceived lower risk associated with these agents, making them more favorable to consumers. These natural compounds are considered safer and help address safety concerns. The antimicrobial agents used in natural packaging can be classified into biologically derived components, including plant extracts, essential oils, bacteriocins, enzymes and many others [92].

Natural extracts have become increasingly valuable in active packaging solutions, as they enable the safe and high-quality delivery of food products. These extracts are now widely used in packaging for both fresh and processed produce. Natural antimicrobial compounds are derived from various sources, including animal tissues such as enzymes, plants like cinnamon, oregano, rosemary, basil and clove for essential oils (EOs), microorganisms including natamycin, nisin and other bacteriocins, as well as organic acids like citric, propionic and sorbic acid. Additionally, organic polymers, fungi and algae are also utilized to extract bioactive compounds from vegetables and fruits, which contribute to the pool of natural bioactive ingredients [101].

- Antimicrobial and antifungal activity of natural plants extracts

Plant extracts offer compelling prospects as ingredients for biodegradable food packaging due to their natural origin and phytochemical properties. These extracts can be utilized to create active materials that enhance shelf life and add value to products. Complex systems derived from plant extracts are frequently employed as active ingredients in food packaging applications. The resulting film, obtained from these natural extracts, is assessed for its contribution to the bioactive and functional characteristics using a dual approach: (1) incorporation of major bioactive compounds at different concentrations, and (2) inclusion of plant extract concentrations equivalent to the concentration of the available bioactive compounds [101].

Amankwaah et al., (2022) [102] demonstrated that, after a 24 h exposure, chitosan films incorporating 15% Green Tea Extracts (GTE) effectively eliminated populations of *E. coli* K12 and *L. innocua* in tryptic soy broth, reducing them to undetectable levels. Furthermore, in a study by Dordevic et al., (2021) [103], which studied the antimicrobial effects of red grape and blueberry extracts, it was shown that the films containing red grape by-products extracts exhibited the highest antimicrobial efficacy against the Gram-negative microorganism *E. coli* CCM 3954. However, the antimicrobial activity of blueberry extracts was not confirmed by the findings. The authors speculate that this might be attributed to the lower concentration of active compounds present in the samples containing by-product extracts compared to those containing extracts from whole fruits/vegetables. Also, the inclusion of turmeric-ethanol extract in chitosan film resulted in an enhanced antimicrobial effect, leading to significant reductions (*p* < 0.05) in the counts of *S. aureus* and *Salmonella* compared to the pure chitosan film. This improvement in antimicrobial activity was observed during a 3 h exposure period [104].

Ekramian et al., (2020) [105] highlighted that the addition of black cumin seed extract to sago film demonstrated notable antibacterial activity against the tested strains of microorganisms, including *S. aureus* and *E. coli*. Furthermore, increasing the percentage of extract resulted in an expansion of the inhibition zone diameter. Specifically, for *E. coli*, the diameter increased from 6.1 ± 0.6 mm to 9.4 ± 0.7 mm, while for *S. aureus*, it increased from 8.3 ± 0.4 mm to 13.6 ± 0.5 mm.

In a study conducted by Nguyen et al., (2022) [106], the investigation of pectin/chitosan (P/CH) films with *Piper beetle* L. leaf (PB) extract demonstrated notable superiority in their effectiveness against *S. aureus*, *B. cereus*, *P. aeruginosa*, and *Klebsiella pneumoniae* compared to the control film. The results indicated that the antimicrobial activity of the P/CH/PB films increased proportionally with the concentration of the PB leaf extract. The authors considered that this enhancement in activity can be attributed to the presence of various phytochemicals in the PB leaf extract, such as carbohydrates, tri-terpenoids, steroids, alkaloids, eugenol, phytol, amino acids, and tannins, all of which act as effective antibacterial agents. Furthermore, the control film made from gelatine, as well as films obtained from chitosan or a blend of chitosan–gelatine, exhibited no effectiveness against any of the Gram-negative strains (*E. coli* and *Shigella sonnei*) and the Gram-positive strains (*B. subtilis* and *L. monocytogenes*). However, the films containing hope extract displayed significant antibacterial activity against the tested strains. Notably, they demonstrated greater effectiveness against Gram-positive bacteria compared to Gram-negative bacteria [107].

In the absence of thyme essential oil (TEO), the composite membrane made from soluble dietary fiber/sodium carboxymethyl cellulose did not exhibit any antibacterial activity. However, when TEO was incorporated into the film, the composite membrane displayed antibacterial properties against *S. aureus* and *E. coli*. But the presence of pectin in the film led to a decrease in the area of the inhibition zone, primarily due to the nutrient-rich environment provided by pectin, which promotes bacterial growth, resulting in a similar area of inhibition zone compared to the film without pectin, indicating a possible interaction between pectin and TEO. Overall, the composite film demonstrated better inhibition against *S. aureus* compared to *E. coli* [108].

Mauro et al., (2022) [97] demonstrated that the inclusion of grape seed oil at a lower concentration (0.5 mL) led to an increase in antimicrobial activity against *S. maltophilia*, *A. guillouiae* and *P. aeruginosa* and no antimicrobial activity against *E. coli* (PSL52), *E. coli* (ATCC 25922), *H. alvei*, *H. paralvei*, *S. typhimurium*, *S. enteritidis* and *En. amnigenus*. On the other hand, the addition of grape seed oil at a higher concentration (1 mL) did not enhance the activity against *E. coli* (PSL52), *S. maltophilia* and *H. alvei*, resulting in a decrease in the inhibition of *E. coli* (ATCC 25922), *H. paralvei*, *S. typhimurium* and *S. enteriditis*, and an increase in the inhibition of *En. amnigenus*. In terms of spoilage bacteria, none of the films exhibited activity against *Pseudomonas poae* and *Pseudomonas endophytica*. Notably, there was evident inhibition against *Pseudomonas lactis* bacteria in the chitosan film and the film with the addition of 0.5 mL grape seed oil, while no inhibition was observed with the film combined with 1 mL grape seed oil.

The antimicrobial effectiveness of collagen/thymol films obtained by Michalska-Sionkowska et al., (2017) [109] was demonstrated against various microorganisms including *E. coli*, *B. subtilis*, *Enterobacter aerogenes*, *Candida albicans*, and *S. aureus*. Among these, *S. aureus* exhibited the highest sensitivity to the antimicrobial properties of thymol.

- Antimicrobial and antifungal activity of essential oils

Polysaccharides and proteins are commonly employed as encapsulation materials for essential oils (EOs) due to their favorable retention and release characteristics. Additionally, these biopolymers are readily accessible at a comparatively affordable cost [110]. The primary reason for the inhibition of both Gram-positive and Gram-negative bacteria by biocomposite films incorporating essential oils is the presence of polyphenolic components. These components play a crucial role in disrupting the biological activity of bacterial cell membranes [95].

Cakmak et al., (2020) [111] demonstrated that different microorganisms may respond differently to essential oils. Bergamot essential oil exhibited higher antimicrobial activity compared to lemon essential oil against both *E. coli* and *S. aureus*, as indicated by larger zones observed around the discs. Lemon oil, although not as potent against *S. aureus*, inhibited the growth of *E. coli*. In the experiments involving *A. niger*, lemon oil did not display significant inhibition. However, it can be inferred that bergamot oil possesses antimicrobial effects on *A. niger*. The essential oils interact with the lipid components of the cell membranes, altering their permeability and leading to the leakage of microbial cells.

In their study, Perdana et al., (2021) [112] investigated the impact of five essential oils (EOs)—lemongrass, kaffir lime peel, guava leaf, plai, and fingerroot—on pathogenic microorganisms. They found that lemongrass essential oil (LEO) had the lowest minimum inhibitory concentration (MIC) against *E. coli* TISTR 512, *S. typhimurium* TISTR 1470, *S. aureus* TISTR 746, *B. cereus* TISTR 035, *C. albicans* TISTR 5554 and *A. niger* TISTR 3130, with values of 1.56 ± 0.00, 0.65 ± 0.23, 0.39 ± 0.00, 0.39 ± 0.00, 0.52 ± 0.18 and 0.78 ± 0.00 μL mL^−1^, respectively. Lemongrass EO also exhibited the most effective minimum bactericidal/fungicidal concentration (MBC/MFC) compared to the other tested EOs. The MIC of LEO against *E. coli* TISTR 512 showed a significant difference (*p* < 0.05) compared to the other EOs. Among the EOs, *M. ruber* TISTR 3006 was found to be the most resistant strain. Guava EO demonstrated the least effectiveness against all tested microorganisms, followed by plai EO. Interestingly, guava EO inhibited *B. cereus* TISTR 035 at a similar level (*p* > 0.05) to lemongrass, kaffir lime and fingerroot, but not plai EO. The authors considered that the two main compounds, α-citral (geranial) and β-citral (neral), exhibited antibacterial properties against both Gram-negative and Gram-positive bacteria, while the third component, myrcene, did not display antibacterial activity on its own but showed activity when combined with either of the other two main components.

The incorporation of black pepper essential oil (BPEO) and ginger essential oil (GEO) into polyvinyl alcohol/gum arabic/chitosan (PVA/GA/CS) films demonstrated significant growth inhibition against *B. cereus*, *S. aureus*, *E. coli* and *S. typhimurium*. Notably, black pepper essential oil exhibited a greater inhibition zone compared to ginger essential oil. The BPEO-PVA/GA/CS composite film displayed the most significant inhibition zones (20.43 ± 2.04 mm, 18.73 ± 1.76 mm, 16.82 ± 1.27 mm, and 17.43 ± 1.36 mm) for *B. cereus*, *S. aureus*, *E. coli* and *S. typhimurium*, respectively, among the composite films. Similarly, the GEO-PVA/GA/CS composite film also exhibited substantial inhibition zones (17.83 ± 1.77 mm, 16.34 ± 1.54 mm, 14.59 ± 1.14 mm, and 15.21 ± 1.74 mm) for *B. cereus*, *S. aureus*, *E. coli* and *S. typhimurium*, respectively [113].

Morsy et al., (2014) [114] demonstrated that the addition of 2% oregano essential oil to pullulan film exhibited activity against *S. aureus* and *S. typhimurium*, while *L. monocytogenes* and *E. coli* O157:H7 were not inhibited. On the other hand, the incorporation of 2% rosemary essential oil in the pullulan film showed activity against *S. aureus*, *L. monocytogenes*, *E. coli* O157:H7 and *S. typhimurium*, compared to the film containing 1% rosemary essential oil. The antimicrobial effect of the essential oils can be attributed to specific compounds such as carvacrol in oregano essential oil and cineole in rosemary essential oil, able to disrupt the bacterial cell membrane. Carvacrol, being hydrophobic, specifically targets the bacterial membrane, depletes the intracellular ATP pool, alters the membrane potential and increases the permeability of the cytoplasmic membrane to potassium ions and proteins.

#### 3.1.2. Bacteriocins

In recent times, there has been a growing interest in biologically derived antimicrobials, particularly due to their effectiveness against *Listeria* bacteria. Bacteriocins, which are peptidic antimicrobial compounds synthesized by various bacteria, have shown bactericidal activity against closely related species. Lactic acid-producing bacteria are known to be the primary producers of bacteriocins, making them highly attractive for controlling specific bacterial growth in food. Incorporating bacteriocins into antimicrobial films can enhance the quality, safety and shelf life of food products. Notably, bacteriocins offer advantages such as thermal stability, hypoallergenic properties and easy degradation by proteolytic enzymes in the gastrointestinal tract [92].

The utilization of nisin in antimicrobial packaging has gained significant attention in recent times. Multiple studies have demonstrated that the incorporation of nisin into antimicrobial films and packages can effectively regulate bacterial growth, thereby preserving food quality, ensuring safety and prolonging the shelf life of food products [92].

The antimicrobial activity of nisin has been observed in various packaging films, including those made of plastic, paperboard and edible materials. It has shown effectiveness against several pathogenic and spoilage microorganisms such as *L. monocytogenes*, *Brochothrix thermosphacta*, *Micrococcus flavus*, *Micrococcus luteus*, *Lactobacillus* spp., *L. innocua*, *S. aureus* and *S. typhimurium*. Moreover, the use of nisin-activated antimicrobial packaging materials has proven successful in extending the shelf life of food products. This has been demonstrated through the application of model food systems such as meat and meat products, milk, cream milk, cheese and orange juice [92].

La Storia et al., (2020) [115] investigated the antimicrobial activity of whey protein/inulin/gelatine (WP) edible films incorporated with bacteriocin-producing lactic acid bacteria (LAB). The findings of the study revealed the effectiveness of these films against *L. innocua* C6, highlighting their potential as an alternative packaging technology for enhancing food safety. Also, the results indicated that WP films containing the *L. curvatus* 54M16 strain showed promising antimicrobial properties, further supporting their potential application in food packaging.

The films obtained by Jimenez-Villeda et al., (2019) [116] from gellan gum, citric pectin, glycerol, CaCl_2_, EDTA and concentrated supernatant (AMC) from *Streptococcus infantarius* fermentations containing bacteriocin-like inhibitory substances, demonstrated notable inhibitory effects against *E. coli*, *S. aureus* and *L. monocytogenes*. Furthermore, per Cao et al., (2019) [117], the addition of nisin (0.2%) to a chitosan (2%) coating has been demonstrated to significantly improve the antimicrobial efficacy for preserving fresh pork.

#### 3.1.3. Biopolymers Enriched with Nanoparticles

Most of the studies have shown that nanoparticles exhibit greater efficacy against Gram-negative bacteria compared to Gram-positive bacteria. This difference in effectiveness can be attributed to the distinct cell wall compositions of these two types of bacteria. Gram-positive bacteria possess an outer layer of peptidoglycan that is thicker and acts as a protective barrier against inhibitory substances. In contrast, Gram-negative bacteria have a thinner peptidoglycan layer in their cell walls [118], which limits diffusion of hydrophobic substances via its lipopolysaccharide membrane that makes them more resistant against antimicrobial agents [95].

The incorporation of metallic nanoparticles (MNPs) into polymers offers a means to extend the shelf life of food products by slowing down enzymatic processes and inhibiting the development of various physiological diseases. The primary focus of metallic nanoparticles (MNPs) is to enhance the functionality of packaging materials, particularly biodegradable films. MNPs such as silver, copper oxide and zinc oxide have been found to significantly improve the performance of biodegradable films due to their large surface area and antimicrobial activity against various microorganisms including fungi, bacteria and molds [119].

The antimicrobial effects of nanoparticles depend on their surface properties, charge, structure, surface-to-volume ratio, nanoscale size and potential synergistic interactions among the nanoparticles. These factors interact with the structural characteristics of bacteria, including their surface charge, to contribute to the overall antimicrobial impact. Metallic nanoparticles can exhibit antimicrobial effects through a three-step process: (1) the release of ions from the nanoparticles; (2) the attachment and penetration of the nanoparticles into the target; and (3) catalysis of active oxygen species and free radical formation [120].

NPs attach to the microbial cell wall and can easily penetrate it, potentially causing damage and leading to the leakage of cytoplasmic content. Moreover, NPs can interact with other cellular structures and biomolecules, including DNA, affecting ATP synthesis and inducing cell apoptosis through DNA damage and lipid peroxidation. Additionally, NPs can interact with biomolecules such as amino and carboxyl groups in the cell wall’s peptidoglycan, generating oxidative stress that disrupts DNA replication and ultimately disrupts the proton motive force across the cell membrane [119].

Extensive studies have verified the remarkable bactericidal properties of ZnO and MgO nanoparticles, with Fe_2_O_3_ nanoparticles exhibiting the least bactericidal activity. The order of antibacterial efficacy was observed as follows: ZnO > MgO > CuO > Fe_2_O_3_ [121].

- ZnO nanoparticles

Zinc oxide nanoparticles (ZnO NPs) possess antimicrobial properties, making them highly valuable for various applications. Studies have shown that within the acidic lysosomal environment, ZnO NPs undergo degradation, leading to the conversion of core metals into ions and the release of toxic substances. This process hampers cell reproduction by interfering with essential cellular functions. Additionally, ZnO NPs exhibit antimicrobial activity through other mechanisms, such as localized microenvironmental changes near the microbes and the generation of reactive oxygen species (ROS). Furthermore, these nanoparticles can enhance the solubility of certain substances, which can disrupt enzymes containing -SH groups within the microbial cells. Consequently, this disruption can cause organelle malfunction, protein denaturation, and DNA damage, ultimately affecting DNA replication in microorganisms [122].

The antimicrobial evaluation conducted on chitosan films and chitosan/nano-ZnO composite films against *E. coli* and *S. aureus* revealed that the composite chitosan films exhibited superior antibacterial efficacy. Specifically, the film incorporating 0.3% of 50 nm zinc oxide particles demonstrated the highest inhibition rate, indicating that smaller-sized nano-ZnO particles possess stronger bacteriostatic activity [123].

Zhang et al., (2017) [124] demonstrated the efficacy of ZnO NPs incorporated into PLA coating in neutralizing both *E. coli* and *S. aureus*. Notably, *E. coli* exhibited higher susceptibility to this particular agent, with a significant 3.14 log reduction observed when the PLA coating layer contained 0.5 wt% of the antimicrobial agent.

The pullulan/collagen/0.5% ZnO NPs composite film demonstrated inhibitory effects against *B. subtilis*, resulting in an inhibition zone of 10 mm. However, the growth of *S. aureus*, *P. aeruginosa* and *C. albicans* was not hindered by the composite film. On the other hand, the composite film displayed antifungal activity against *A. niger*, with the maximum inhibition zone of 18 mm. On the contrary, the presence of 0.25% ZnO NPs in the composite film did not exhibit antifungal activity, which means that the antifungal activity decreased as the nano ZnO concentration increased to 1%. One possible explanation for this observation could be the formation of nanoclusters of a larger size within the polymer matrix. It has been suggested that ZnO-NPs may impact fungal cell functions by increasing the nucleic acid content, which could be a result of the stress induced by the presence of nanoparticles within the fungal cells [125].

Zinc oxide nanoparticles with a size of 110 nm exhibited higher activity against *S. aureus*, *L. monocytogenes*, *E. coli* O157:H7 and *S. typhimurium*, in comparison to ZnO nanoparticles with sizes of 100 nm or 130 nm. On the other hand, Ag NPs with a size of 100 nm demonstrated higher activity against *S. aureus* than *L. monocytogenes* [114].

The pullulan/collagen/ZnO-NPs (0.5%) composite films obtained by Bailore et al., (2020) [125] demonstrated effective activity against *A. niger*, with a maximum zone of inhibition measuring 18 mm. These films also exhibited inhibitory effects on the Gram-positive bacterium *B. subtilis*, showing a zone of inhibition of 10 mm. On the contrary, the growth of *S. aureus*, *P. aeruginosa* and *C. albicans* was not inhibited by the obtained films.

All these findings highlight the potential of ZnO nanoparticles as an effective antibacterial additive ingredient in biopolymer films and composites for food pathogen inhibition.

- Fe nanoparticles

Fe_3_O_4_ (magnetite) and γ-Fe_2_O_3_ (maghemite) are magnetic iron oxide nanoparticles (MNPs) that are widely used due to their favorable properties. However, these MNPs differ in terms of their iron oxidation states and structures, which in turn affect their overall physicochemical characteristics. Additionally, there are other types of magnetic nanoparticles with similar promising properties, such as titanomagnetite and cobalt- or nickel-based magnetic materials. Iron magnetic nanoparticles possess a high surface-area-to-volume ratio, which gives them unique qualities compared to bulk materials, including a lower melting point, lower sintering temperature and distinctive magnetic properties [126].

The incorporation of Fe_3_O_4_ magnetic nanoparticles into chitosan–pectin films resulted in improved antimicrobial activity against *E. coli* and *S. epidermidis* bacteria. This suggests that chitosan–pectin films with Fe_3_O_4_ magnetic nanoparticles hold great potential for applications in active and intelligent food packaging [118].

The pure gelatin films did not exhibit any inhibition against *E. coli* and *S. aureus*. However, after incorporating magnetic iron oxide (MIO) NPs into gelatin, inhibition zones were observed against tested strains. The gelatin nanocomposite films with 20% MIO NPs demonstrated significantly greater inhibition against *S. aureus* (8.22 ± 1.04 mm) and *E. coli* (7.10 ± 0.08 mm) [127].

The antibacterial and antifungal activity of Fe_3_O_4_ NPs, both in their bare form and coated with chitosan, was evaluated against five organisms: *E. coli*, *B. subtilis*, *C. albicans*, *A. niger* and *Fusarium solani*, by Nehra et al., (2017) [128]. The results showed that the chitosan-coated iron oxide nanoparticles exhibited a significant effect on the organisms tested, with the following ranking: *F. solani*/*A. niger* < *C. albicans* < *E. coli*/*B. subtilis* (*p* < 0.001).

- TiO_2_ nanoparticles

Over the past decade, titanium dioxide (TiO_2_) has emerged as a commonly used three-dimensional (3D) nanoparticle for enhancing packaging materials. TiO_2_ is a cost-effective and non-toxic nanoreinforcement that exhibits effective bactericidal activity against various foodborne microorganisms and allergens. One notable feature that sets TiO_2_ nanoparticles (TiO_2_ NPs) apart from other metal oxide NPs is their remarkable photocatalytic activity [129].

TiO_2_ NPs generate reactive oxygen species (ROS), which can disrupt the integrity of the bacterial outer membrane and compromise its overall functionality. The direct interaction between TiO_2_ NPs and bacterial cells results in the impairment of the cell wall or membrane, leading to the release of intracellular components. Moreover, the chitosan-TiO_2_ composite coatings directly target intracellular substances, triggering the production of oxygen free radicals (OH and O^2−^) that attack the outer membrane. This oxidative assault causes DNA damage, disruption of ribosome function, interference with electron transport processes, and oxidation or degradation of bacteria, ultimately resulting in bacterial death [130].

The chitosan–TiO_2_ composite films exhibit enhanced antimicrobial activity against *E. coli* and *S. aureus* compared to the chitosan coating used as the control film, which displayed some antibacterial activity against *E. coli* (9.86 ± 0.90 mm) and *S. aureus* (12.13 ± 0.48 mm). The antimicrobial activity observed in the pure chitosan coating films may be attributed to amino protonation and subsequent cationic production, facilitated by the ultra-long molecular chain of chitosan, which enables effective binding to *E. coli* and *S. aureus*. As the concentration of TiO_2_ NPs was increased, the size of the inhibition zone exhibited by the composite coating against *E. coli* and *S. aureus* increased in comparison to the control group. At a concentration of 0.05% and 0.09% of TiO_2_ NPs, the maximum inhibition zones observed for *E. coli* and *B. cereus* were 11.37 ± 0.76 mm and 0.55 ± 0.35 mm, respectively, which were significantly different from those of the control group (*p* < 0.05). These values indicate a pronounced bacteriostatic effect on the tested strains at these concentrations [130]. Also, Ulu et al., (2020) [131] reported that chitosan/TiO_2_ NPs films exhibited antimicrobial activity against *E. coli*, *S. aureus* and *C. lipolytica*. The authors attributed the antimicrobial efficacy to the interaction between the NH^3+^ groups of chitosan and the negatively charged cell membrane, resulting in the leakage of cytoplasmic contents into the extracellular matrix.

- Ag nanoparticles

Inorganic nanoparticles, particularly silver nanoparticles (Ag NPs), are widely utilized as antimicrobial agents. Ag NPs have exhibited potent antimicrobial activity surpassing that of silver ions (Ag^+^). Nevertheless, concerns have arisen regarding their cytotoxicity. The exact mechanisms of toxicity, long-term accumulation effects, and the dose–response relationship of Ag NPs remain significantly unclear [132].

The precise mechanism underlying the antibacterial activity of Ag NPs remains poorly understood, but is considered that Ag NPs carry a positive charge, facilitating their interaction with the negatively charged peptidoglycan cell wall of bacteria through electrostatic interactions. This interaction between Ag NPs and the bacterial cell membrane hinders DNA replication and cell division, leading to a decline in mesosomal function and an elevation in the generation of reactive oxygen species (ROS). In case of fungi, Ag NPs have the capability to create transmembrane pores in the fungal cell membrane, resulting in cell damage by obstructing nutrient intake. Additionally, they can bind to DNA molecules and induce chromosomal aberrations. These chromosomal aberrations have the direct effect of disrupting fungal cell division and triggering fungal cell apoptosis [133].

The incorporation of Ag NPs into Furcellaran films resulted in antimicrobial activity against a range of bacteria and fungi, of which *P. aeruginosa*, *E. faecalis* and *S. aureus* were particularly susceptible, exhibiting significant growth inhibition as determined by the disc diffusion method [134].

In a study conducted by Kalaivani et al., (2018) [135], it was demonstrated that chitosan/Ag NPs exhibited antibacterial activity against various bacterial species. *Bacillus* sp. and *Staphylococcus* sp. showed inhibition zones of 15 mm and 13 mm, while *Pseudomonas* sp. exhibited a larger inhibition zone of 24 mm, followed by *E. coli* with an inhibition zone of 18 mm. *Proteus* sp., *Serratia* sp. and *Klebsiella* sp. displayed inhibition zones of 12 mm, 14 mm and 15 mm, respectively. Regarding fungal pathogens, *A. niger* showed the largest inhibition zone with a diameter of 15 mm, followed by *A. fumigatus* and *A. flavus* with inhibition zones of 13 mm each, and *C. albicans* with an inhibition zone of 11 mm.

The results obtained by Jung et al., (2018) [133] demonstrated that all the chitosan/starch/Ag NP-coated papers have good antibacterial activity against *E. coli* and *S. aureus* bacteria, compared to uncoated paper and starch- and chitosan-coated papers. The chitosan/starch/Ag NP-coated papers exhibited various inhibition zones, such as, *E. coli* (9:1 = 1.7 mm, 8:2 = 1.8 mm, 7:3 = 2.0 mm and 5:5 = 2.2 mm) and *S. aureus* (9:1 = 1.5 mm, 8:2 = 1.5 mm, 7:3 = 2.0 mm and 5:5 = 2.0 mm), depending on the bacteria and composition of starch. Also, Anvar et al., (2021) [136] revealed the remarkable effectiveness of the chitosan/Ag NP nanocomposite film against microorganisms such as *E. coli*, *B. subtilis*, *P. aeruginosa* and *S. aureus*. Similarly, alginate/Ag NP nanocomposite films demonstrated the inhibitory effect of silver nanoparticles against *S. aureus* and *E. coli* [137]. Overall, Ag NPs demonstrated promising antimicrobial activity against both bacterial and fungal pathogens.

- Au nanoparticles

Gold nanoparticles (Au NPs) hold great promise in the development of antibacterial agents owing to their favorable characteristics, including low toxicity, high potential for functionalization, versatile effects, convenient detection, and photothermal activity [132]. The antimicrobial activity of Au NPs is greatly influenced by their size and shape, allowing them to effectively target a wide range of microorganisms [136].

The antifungal activity of chitosan–gold nanoparticles (Chi/Au NPs) against pathogenic against *C. albicans*, *A. terreus*, *A. niger* and *A. fumigatus* was assessed by Hashem et al., (2022) [138]. The results demonstrated that Chi/Au NPs exhibited remarkable antifungal activity against both single-celled and multicellular fungi, but stronger antifungal effects against single-celled fungi compared to multicellular fungi. For instance, the inhibition zone of Chi/Au NPs was 25 mm, 20 mm, 22 mm, and 23 mm against *C. albicans*, *A. terreus*, *A. niger* and *A. fumigatus*, respectively. In contrast, Au^+^ demonstrated weak antifungal activity against *C. albicans* and *A. terreus*.

The addition of Au NPs to PVA–glyoxal and PVA–glutaraldehyde (GA) films enhanced their antimicrobial activity, resulting in a 13 mm inhibition zone against *E. coli*, compared to the inhibition zone of 10 mm revealed by PVA–GA–graphene oxide (GO) composite film. The antimicrobial activity of the films was attributed to both graphene oxide and Au NPs, with Au NPs exhibiting higher antimicrobial capability compared to GO [139].

The antibacterial characteristics of the chitosan/Au NPs were assessed against *S. aureus* and *E. coli*. The results demonstrated that the addition of Au NPs resulted in a substantial enhancement of the zone of inhibition of tested strains. Moreover, the composite materials exhibited a higher inhibition rate against *E. coli* compared to *S. aureus* [140].

- MgO nanoparticles

Magnesium oxide (MgO) nanoparticles are highly valued in various industries due to their exceptional physicochemical properties, making them environmentally friendly, economically viable, and industrially significant. These properties include remarkable refractive index, excellent corrosion resistance, high thermal conductivity, low electrical conductivity, physical strength, stability, flame resistance, dielectric resistance, mechanical strength, and exceptional optical transparency [141]. Due to their structural characteristics, surface properties, and stability, MgO nanoparticles show significant promise as antimicrobial agents in the field of food safety applications [142]. The strong antimicrobial activity of MgO nanoparticles can be attributed to the release of reactive oxygen species (ROS), which attack the bacterial cell membrane, causing leakage and ultimately leading to bacterial death. Additionally, when MgO comes into contact with water, it forms an aqueous solution of Mg(OH)_2_, resulting in pH alteration and causing extensive bacterial death [143].

The PLA/MgO film revealed superior antibacterial efficacy, because the addition of 2 wt% MgO NPs led to progressive damage and death of approximately 46% of the *E. coli* bacterial culture after a 12 h treatment, in contrast to control PLA film [144].

The carboxymethyl chitosan (CMCS) control film without MgO NPs displayed moderate antimicrobial activity against *L. monocytogenes* and *S. baltic*, with inhibition ratios of 80% and 39.2%, respectively. However, the incorporation of MgO nanoparticles significantly enhanced the antibacterial efficacy of the CMCS film. At a 1% MgO content in the composite film, the inhibition rate further increased, surpassing 99.99% for both *L. monocytogenes* and *S. baltic*. These results highlight the improved antibacterial performance of the CMCS composite film, indicating its potential for broader applications in the field of food packaging [143].

- Cu nanoparticles

Copper nanoparticles (Cu NPs) have attracted significant attention due to their mechanical, electrical, magnetic and thermal properties. They have found applications in various fields such as water treatment, heat transfer systems and antimicrobial coatings and films [145]. Studies demonstrated that Cu NPs synthesized using various copper salts exhibited antimicrobial activity against both *L. monocytogenes* and *E. coli*, two common foodborne pathogens [136]. It has been reported in various studies that Cu NPs exhibit fungicidal properties against a wide range of plant fungi, like *Fusarium* sp., *Phoma destructiva*, *Curvularia lunata*, *Alternaria alternate*, *Fusarium oxysporum*, *Penicillium italicum*, *Penicillium digitatum* and *Rhizoctonia solani* [146].

After silver, copper is one of the most frequently utilized nanomaterials because of its low cost and widespread availability. However, its synthesis poses challenges due to copper’s high susceptibility to oxidation. Copper readily reacts with air, leading to the formation of thermodynamically stable oxidized forms [132]. Akturk et al., (2020) [147] synthesized Cu NPs with soluble starch (CuS NPs) and sodium alginate (CuA NPs) for testing the antimicrobial and antifungal efficacity. Alginate films incorporated with CuS NPs exhibited antibacterial activity, which varied depending on the type of bacteria and the concentration of CuS NPs, in comparison to neat alginate film. The composite films composed of alginate and CuS NP demonstrated stronger antibacterial activity against *E. coli* compared to *L. monocytogenes*. It was evident that the antibacterial activity increased with higher concentrations of CuS NPs against both types of bacteria. In the case of *E. coli*, the composite film exhibited a bactericidal effect at a low CuS NP concentration (0.5 wt%), while at a higher concentration (1.5 wt%), it demonstrated a strong bactericidal effect leading to complete eradication of the bacteria after 12 h of incubation. Conversely, the alginate/CuS NP composite film only exhibited a growth-delaying effect on Gram-positive bacteria (*L. monocytogenes*). In case of fungal activity, CuS NPs and CuA NPs tested against *C. albicans* (ATCC 10231) and *C. krusei* (KUEN 1001) showed inhibitory activity after 16 h and the inhibitory activity was increased with the increase in the concentration of Cu NPs [115]. Also, Roy and Rhim (2020) [148] demonstrated that the antibacterial activity of alginate/CuS NP composite films was more pronounced against *E. coli* compared to *L. monocytogenes*. The antimicrobial efficiency was higher than that obtained for neat alginate film. The antibacterial activity was directly proportional with CuS NP concentration against both types of bacteria. For *E. coli*, the composite film exhibited bactericidal effects at a lower CuS NP concentration (0.5 wt%), while at a higher concentration (1.5 wt%), it displayed a strong bactericidal effect that completely eradicated the bacteria after 12 h of incubation. Conversely, the alginate/CuS NP composite film only exhibited a growth-delaying effect on the Gram-positive bacteria (*L. monocytogenes*).

The same authors revealed that gelatine film incorporating copper sulfide NPs (CuS NPs) showed a significant reduction in cell viability for *E. coli*, while it did not have a significant impact on the growth of *L. monocytogenes*. At a low concentration (0.5 wt%), CuS NPs did not exhibit sufficient inhibitory activity against microbial growth. However, at a high concentration (2.0 wt%), they demonstrated strong antibacterial activity, effectively suppressing the growth of *E. coli* completely within 9 h of contact [149]. In case of agar film, the control film did not exhibit any antibacterial activity against *E. coli* and *S. aureus*, compared to agar/CuS NP composite film which had more pronounced antibacterial activity against *E. coli* than *L. monocytogenes* [150].

In case of chitosan films, the study conducted by Shankar et al., (2018) [151] showcased the enhance of antimicrobial efficacy in chitosan films through the incorporation of CuS NPs, effectively inhibiting the growth of foodborne pathogenic bacteria and preventing contamination.

The agar/CuS NP film obtained by Levard et al., (2013) [152] exhibited lower antibacterial activity compared to the agar/Cu NP film, primarily attributed to the diminished antimicrobial properties resulting from sulfation of copper. Nevertheless, sulfidation of copper presents a benefit in biological applications as it reduces the cytotoxic effects associated with copper.

#### 3.1.4. Synergistic Action of Different Antimicrobial Agents

Combining two or more substances in synergistic approaches can yield superior efficacy compared to individual substances alone. One promising approach is the conjugation of magnetic nanoparticles (MNPs) with other antimicrobial compounds, which has the potential to enhance their effectiveness [132], or combination of different antimicrobial agents in biopolymer films or coatings, like nisin and essential oils [95], coconut essential oil, paprika extract and NPs [153].

For this purpose, Anaya-Esparza et al., (2019) [154] demonstrated that TiO_2_-ZnO-MgO mixed oxide nanomaterials (MON) exhibited enhanced antibacterial properties against *E. coli*, *S. paratyphi*, *S. aureus* and *L. monocytogenes* compared to undoped TiO_2_ NPs. The antibacterial activity of the mixed oxide nanomaterials followed this order: *E. coli* > *S. paratyphi* > *S. aureus* > *L. monocytogenes*. While the exact mechanism of action of MON against bacteria is not fully understood, the authors consider that oxidative stress induced by the generation of reactive oxygen species (ROS) may lead to lipid peroxidation of the cell wall, affecting membrane fluidity, disrupting cell integrity, promoting the release of intracellular contents and, ultimately, causing bacterial cell death.

Bui et al., (2018) [155], tested the efficacity of composite films consisting of soy protein isolate/Ag NPs/platelike aminoclay against *E. coli* and *S. aureus*. The growth of *E. coli* was more effectively inhibited compared to *S. aureus*, resulting in diameters ranging from 14.9 to 17.1 mm, depending on the loading amount of aminoclay. Notably, the diameter significantly increased as the aminoclay (AC) content increased from 5 to 8 wt%. However, a further increase in the AC content from 8 to 14 wt% did not significantly affect the diameter. The increased antibacterial activity of the soy protein isolate/Ag NPs/platelike aminoclay composite film can be attributed to the electrostatic force generated by the protonated groups (R-NH_2_) present on the aminoclay lamella, driving the antimicrobial effect.

Thirumurugan et al., (2013) [156], demonstrated that the antimicrobial activity against four food-spoiling organisms, namely *Micrococcus luteus*, *B. cereus*, *S. aureus* and *E. coli*, was enhanced when Au NPs were combined with bacteriocin.

In their study, Artfat et al., (2017) [157] investigated the antimicrobial activity of agar/Ag-Cu nanoparticle films against *L. monocytogenes* and *S. typhimurium*. The nanocomposites demonstrated strong inhibitory effects against both organisms. The antimicrobial action of Ag-Cu nanoparticles against these bacteria could be attributed to the release of silver and copper ions. These ions may penetrate the gaps and pits in the bacterial membrane, interact with sulfhydryl or disulfide groups of enzymes, or attach to the negatively charged bacterial cell wall. These interactions can disrupt metabolic processes and lead to the rupture of the bacterial cell wall, ultimately causing cell death.

The findings of Akhter et al., (2019) [95] indicated a notable enhancement in the antimicrobial activity of the biocomposite film comprising chitosan/pectin/starch/0.5% rosemary oil/nisin. This enhanced activity was observed across all tested strains, with a particularly pronounced increase in the inhibition zone against Gram-positive bacteria such as *B. subtilis* and *L. monocytogenes*, compared to the Gram-negative bacteria *E. coli*.

In comparison to sodium alginate/TiO_2_ NPs film, the antimicrobial activity of sodium alginate/Au-TiO_2_ nanocomposites film exhibited a significant improvement, showing a 60% increase against *S. aureus* and a 50% increase against *E. coli* [158].

Asdagh et al., (2021) [153] demonstrated that the antimicrobial activity against *S. aureus* and *E. coli* was enhanced in whey protein isolate films incorporated with 0.8% coconut essential oil and 0.06% paprika extract and CuO nanoparticles, compared to whey protein isolate films containing only 0.8% coconut essential oil or 0.06% paprika extract in combination with CuO nanoparticles.

Arezoo et al., (2020) [159] investigated the effects of incorporating cinnamon essential oil (CEO) and TiO_2_ NPs into sago starch films on the growth of *S. typhimurium*, *E. coli* and *S. aureus*. The results revealed that the controlled sago starch films without TiO_2_ NPs or CEO did not exhibit any inhibition zones, indicating no antimicrobial activity. However, the inclusion of both TiO_2_ NPs and CEO significantly increased the size of the inhibition zone, which suggest that sago starch/CEO/TiO_2_ NPs possess antimicrobial activity against the tested microorganisms. Cinnamaldehyde, which is the main active component of CEO, is known for its natural antimicrobial properties. The hydrophobic nature of CEO contributes to its ability to destabilize and disrupt bacterial membranes, and when combined with TiO_2_, it results in cytoplasmic leakage and eventual destruction of bacterial cells.

The gelatin/κ-carrageenan films demonstrated no bacteriostatic properties, but the films containing TiO_2_ exhibited a significant inhibition zone for both bacteria, *S. aureus* and *E. coli*, indicating their effective antimicrobial activity. Furthermore, the incorporation of anthocyanins into the films resulted in an increase in the diameter of the inhibition zone, suggesting that they also possessed antimicrobial properties. The antimicrobial mechanisms of TiO_2_ and anthocyanins may involve increasing cell membrane permeability and interacting with crucial cellular components such as phospholipids, lipids, proteins and nucleic acids. Anthocyanins are known to contain phenolic compounds that exhibit notable antimicrobial activity. The bacteriostatic properties of the films were more pronounced against *S. aureus* compared to *E. coli* (*p* < 0.05), which could be attributed to differences in the cell wall structures of these two bacteria [160].

In the study by Chowdhurry et al., (2020) [139], it was noted that PVA film exhibited no antimicrobial activity, as indicated by the absence of an inhibition zone. In contrast, the PVA–glyoxal and PVA–glutaraldehyde (GA) films displayed inhibition zones of 10 mm and 8 mm, respectively, suggesting moderate antibacterial activity. This antibacterial effect can be attributed in part to the presence of ethanol in the PVA-crosslinked film, which increase the water solubility of the outer protective cell membrane (lipid) of bacteria, leading to the loss of structural integrity and disintegration of the cell membrane. As the cell membrane disintegrates, ethanol can enter the cell and denature proteins, ultimately causing bacterial death. The same study demonstrated that by addition of Au NPs into the PVA–glyoxal film, the antimicrobial activity was further enhanced, resulting in a 13 mm inhibition zone, indicating strong antimicrobial activity. Additionally, the PVA–GA–graphene oxide (GO) film exhibited bacterial cytotoxicity, forming a 10 mm inhibition zone against *E. coli*, which can be classified as moderate antibacterial activity. The bacterial cytotoxicity of the PVA–GA–GO film can be attributed to the insertion of GO nanosheets into the cell membrane.

The inclusion of ZnO nanoparticles resulted in a notable enhancement of the antimicrobial activity in the chitosan film compared to the control film. However, when the antioxidant of bamboo leaves (AOB) was incorporated, the antimicrobial property of the CS/ZnO film continued to increase, suggesting that AOB exhibited a synergistic effect with ZnO nanoparticles in improving the antimicrobial activity of the chitosan film against *S. aureus* and *E. coli* bacteria [161].

The pure soluble soybean polysaccharide (SSPS) films displayed no antimicrobial activity against *P. aeruginosa* and *S. aureus*. However, the incorporation of curcumin into SSPS/TiO_2_ films effectively inhibited the growth of the tested bacteria. Notably, the greatest inhibitory effect was observed on *S. aureus*, with an inhibition zone area of 391.2 mm^2^, followed by *P. aeruginosa* with an area of 290.3 mm^2^. The inhibition zones for pure SSPS, SSPS/TiO_2_, SSPS/TiO_2_-0.2% curcumin, and SSPS/TiO_2_-0.6% curcumin composites against *S. aureus* were measured 0, 25.3, 110.1, and 290 mm^2^, respectively. These results indicate a synergistic effect between curcumin and TiO_2_ nanoparticles. The antibacterial activity of curcumin is attributed to its interaction with membrane proteins of bacterial cells, leading to the inhibition of bacterial growth [162].

## 4. Conclusions

Foodborne pathogens that cause illnesses represent a current cause for concern worldwide, both from human health and economic loss points of view. These problems could be solved by various applications in the food industry related to preservation methods able to control these pathogens. This study describes various emerging technologies with applications in the food industry for solid, liquid and even packed food product preservation.

The emerging technologies discussed in this study proved to be efficient in the total inactivation or reduction in microbial load in products from different categories (both anima and vegetal based products). However, some limitations may occur related to the preservation of the product’s quality, limitations that can be passed through more research of these technologies and application on a wider variety of food products. Food packaging materials research has seen a great increase in the last few years, the producers being preoccupied with obtaining packaging materials with antimicrobial properties, while using biobased materials and ingredients for the developed materials to be environmentally friendly. As discussed here, the potential of reduction in microbial pathogens from different food products by using antimicrobial packaging is promising, the only limitations of these materials being related to fulfilling the characteristics (mainly physical-mechanical) reached by conventional materials. The different antimicrobial agents incorporated in packaging materials showed potential in inhibiting both Gram-positive and Gram-negative bacteria as well as different fungi and yeasts.

The results presented in this revision show the potential of different emerging technologies and packaging materials to inhibit pathogenic microorganisms, which could be responsible for foodborne outbreaks if the food becomes contaminated along the food supply chain. Further research could refer to ingredients used in food production to inhibit the growth and development of microorganisms, as well as a study regarding different methods for surface decontamination in food industry, which could be useful to prevent food contamination. Another line of focus for future research could be a study on the physical-mechanical characteristics of novel packaging materials in comparison with the conventional ones, in terms of resistance, compatibility, migration properties, etc.

## Data Availability

Not applicable.
